# Cerebellar Cytokine Expression in a Rat Model for Fetal Asphyctic Preconditioning and Perinatal Asphyxia

**DOI:** 10.1007/s12311-014-0559-2

**Published:** 2014-04-27

**Authors:** Evi Vlassaks, Tomasz Brudek, Bente Pakkenberg, Antonio W. D. Gavilanes

**Affiliations:** 1 Department of Pediatrics, Division of Neonatology, School of Mental Health and Neuroscience (MHeNS), Maastricht University Medical Center, Maastricht, The Netherlands; 2 Research Laboratory for Stereology and Neuroscience, Bispebjerg University Hospital, Copenhagen, Denmark

**Keywords:** Cerebellum, Cytokines, Fetal asphyctic preconditioning, Perinatal asphyxia, Rat

## Abstract

Asphyctic brain injury is a major cause of neuronal inflammation in the perinatal period. Fetal asphyctic preconditioning has been shown to modulate the cerebral inflammatory cytokine response, hereby protecting the brain against asphyctic injury at birth. This study was designated to examine the effects of perinatal asphyxia and fetal asphyctic preconditioning on the inflammatory cytokine response in the cerebellum. Fetal asphyxia was induced at embryonic day 17 by clamping the uterine vasculature for 30 min. At term birth, global perinatal asphyxia was induced by placing the uterine horns in saline for 19 min. Pro- and anti-inflammatory cytokine expression were assessed by real-time PCR and immunohistochemistry in cerebella of newborn rats. We found that tumor necrosis factor alpha and interleukin-10 mRNA were increased 12 h after fetal asphyxia, while the inflammatory cytokine response was decreased 96 h postfetal asphyxia. When applied as preconditioning stimulus, fetal asphyxia attenuates the cerebellar cytokine response. These results indicate that sublethal fetal asphyxia may protect the cerebellum from perinatal asphyxia-induced damage via inhibition of inflammation.

## Introduction

Perinatal asphyxia (hypoxia–ischemia), affecting 2–4 neonates per 1,000 births, is a major birth concern. Almost every organ of the body can be affected by asphyxia, which may lead to multi-organ failure. However, if affected, the most vulnerable organ is the central nervous system [1]. Infants affected by global asphyxia may develop hypoxic-ischemic encephalopathy (HIE) with neurodevelopmental abnormalities in childhood, presenting as learning disability, seizure activity, motor impairment, and, in the most severe cases, death [2, 3]. Many of these deficits may be related to cerebellar injury, since the cerebellum is one of the brain regions playing an important role in the control of motor functions but also in higher cognitive functions and behavioral changes [4]. Also, the cerebellum is particularly vulnerable in newborn babies because of its rapid growth and development in the third trimester of pregnancy [2, 5]. Moreover, previous studies have shown that cerebellar lesions caused by perinatal asphyxia greatly affect locomotor activity [6, 7].

Recent animal experiments have shown that fetal asphyctic preconditioning, induced by a brief episode of experimental hypoxia–ischemia, offers neuroprotection against perinatal asphyxia [8, 9]. Moreover, we have shown that the inflammatory cytokine expression might play an important role in this preconditioning phenomenon [10]. As knowledge of these pathways is of outmost importance for the development of neuroprotective strategies, we examined whether inflammatory cytokine expression was increased in cerebella of rats that had experienced perinatal asphyxia during Caesarean section birth, and, if so, whether fetal asphyctic preconditioning can modulate this inflammatory response in the cerebellum.

## Materials and Methods

### Animals and Experimental Design

All experiments were approved by the Animal Ethics Board of Maastricht University on animal welfare according to Dutch governmental regulations. Timed-pregnant Sprague–Dawley rats (E14; Charles River, France) were kept under standard laboratory conditions (food and water given ad libitum, 21 ± 2 °C environment temperature, and a 12 h light/dark schedule). Unsexed fetuses and male neonates were used within this study.

**Fig. 1:**
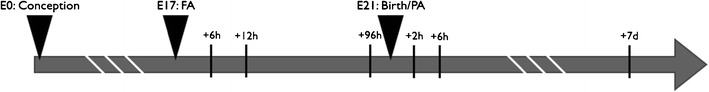
Experimental design. Fetal asphyxia (FA) was induced at E17 by clamping the uterine vasculature during 30 min. At term birth, global perinatal asphyxia (PA) was induced by placing the uterine horns containing the pups in a saline bath (37 °C) for 19 min. All animals were delivered by Caesarean section. Pups were euthanized at 6, 12, and 96 h after FA prenatally (*n* = 6 per group per time point) and at 2 h, 6 h, and 7 days postnatally (*n* = 6 per group per time point). *E* embryonic day, *FA* fetal asphyxia, *PA* perinatal asphyxia

Fetal asphyxia (FA) and perinatal asphyxia (PA) were induced as previously described by Vlassaks et al [10]. FA was induced at embryonic day 17 (E17) by clamping both uterine and ovarian arteries for 30 min. Thereafter, reperfusion was permitted by detaching the clamps, the uterine horns were placed back intra-abdominally, and the abdominal cavity was closed. At E21/22, PA was induced. Pregnant rats were killed by decapitation to avoid the potential effect of the anesthetic. After hysterectomy, the uterine horns containing the pups were placed in saline for exactly 19 min. Then, the pups exposed to PA were delivered and stimulated manually to breathe in a closed incubator. The effect of preconditioning (PC) was studied by inducing FA at E17, followed by PA at E21/22. All pups, control and asphyxiated, were born by Caesarian section (C section). Pups were randomly cross-fostered with surrogate dams (maximally 12 pups each dam), which had given birth vaginally on the same day. Animals were sacrificed at 6, 12, and 96 h after the FA insult and 2 h, 6 h, and 7 days after birth (*n* = 6 for each group at every time point; Fig. 1). Total cerebella of fetal and neonatal pups were collected and snap-frozen. Additionally, right hemispheres (cerebrum + cerebellum) of postnatal brains were fixated in somogyi, glutaraldehyde, and different sucrose concentrations prior to cryopreservation to ensure proper tissue fixation for immunohistochemistry. All samples were stored at −80 °C prior to further analysis.

### RNA Isolation

Total RNA was extracted from frozen cerebellum using the GeneJET RNA Purification Kit (Thermo Scientific) according to the manufacturer’s guidelines. DNase treatment was performed with the Turbo DNA-free kit (Applied Biosystems). RNA integrity and quantity were determined using the Agilent 2100 Bioanalyzer (Agilent Technologies), and only samples with RIN ≥8 were used for further analyses. The RNA samples were stored at −80 °C until use.

### Real-time PCR

Primers for interleukin (IL)-1β, tumor necrosis factor alpha (TNF-α), IL-10, and IL-6 (Table 1) were designed using Oligo software ver.7. Real-time PCR reactions were carried out using Brilliant II SYBR Green QRT-PCR Low ROX Master Mix (Agilent Technologies; 40 cycles—20 s at 95 °C, 15 s at 60 °C, and 15 s at 72 °C). All samples were analyzed in duplicate. Samples negative for RevertAid Reverse Transcriptase were used as appropriate control to ensure specific amplification and to check for genomic DNA contaminations. The real-time PCR was performed on an Mx3005P Real Time PCR cycler (Agilent Technologies). A comparative cycle of threshold fluorescence (Ct) method was used. The relative transcription level of the target gene was normalized to that of GAPDH (primer sequence given in Table 1) and expressed as relative quantity to the calibrator sample using the Pfaffl method [11].

**Table 1:** Rat-specific primers designed for RT-PCR analysis

Gene	Accession number	Primer	Tm°	Sequence (5′-3′)
GAPDH	NC_005103.3	GAPDH for	63.4	CTCCCATTCTTCCACCTTTG
		GAPDH rev	63.8	ATGTAGGCCATGAGGTCCAC
IL-1β	NM_031512.2	IL-1β for	67.0	TACCTATGTCTTGCCCGTGGAG
		IL-1β rev	67.6	ATCATCCCACGAGTCACAGAGG
TNFα	NM_012675.3	TNFα for	64.0	TGCCTCAGCCTCTTCTCATT
		TNFα rev	63.2	GGGCTTGTCACTCGAGTTTT
IL-6	NM_012589.2	IL-6 for	62.5	AAAGCCAGAGTCATTCAGAGC
		IL-6 rev	63.8	GAGCATTGGAAGTTGGGGTA
IL-10	NM_012854.2	IL-10 for	64.0	CCTGCTCTTACTGGCTGGAG
		IL-10 rev	63.8	TGTCCAGCTGGTCCTTCTTT

### Immunohistochemistry

Fixed brain hemispheres of postnatal rat pups were cut into 16-μm-thick sagittal sections. Inflammatory cytokine expression was determined by immunohistochemical staining for IL-1β, TNF-α, IL-6, and IL-10. After antigen retrieval with citrate buffer, endogenous peroxidase activity was blocked by incubation in 3 % hydrogen peroxide. Nonspecific binding sites were blocked with 5 % bovine serum albumin. Cytokines were stained with rabbit polyclonal anti-IL1β (Santa Cruz Biotechnology, sc-7884, dilution 1:200), goat polyclonal anti-TNFα (Santa Cruz Biotechnology, sc-1350, dilution 1:500), goat polyclonal anti-IL6 (R&D Systems, AF506, dilution 1:500), and goat polyclonal anti-IL-10 (Santa Cruz Biotechnology, sc-1783, dilution 1:100). Envision System horseradish peroxidase (HRP) antirabbit (DAKO K4003) and polyclonal rabbit antigoat HRP (DAKO P0449, dilution 1:200) were applied as secondary antibodies. Staining was performed with DAB, and sections were counterstained with cresyl violet. Sections were photographed using Nikon Eclipse 80i microscope and Visiopharm Integrator System software.

### Statistical Analysis

All results are expressed as mean ± standard error of the mean (SEM). Statistical analyses were conducted using GraphPad Prism version 5.0. Comparisons between prenatal control and FA animals were analyzed using Student’s *t* test. All postnatal data were analyzed using one-way analysis of variance (ANOVA) test, followed by post hoc comparisons using LSD correction. *p* < 0.05 was considered statistically significant.

## Results

Since asphyxia is an evolving process, the aim was to investigate the effects of asphyxia both at early and later time points. In previous studies, we showed that asphyxia could induce early inflammatory changes in the brain [10, 12]. On this ground, the time points 6/12 h post-FA and 2/6 h post-PA were chosen (Fig. 1). As a later time point, we chose 96 h after FA since at this stage the pups are primed to be born and thus presumably are “preconditioned”. Additionally, we included 7 days after birth (Fig. 1) because at this time point we have previously demonstrated in our model that apoptosis is increased after severe PA compared with preconditioned animals [8].

### Increased TNF-α and IL-10 mRNA levels 12 h after FA, but Decreased Cytokine Levels at 96 h Post-FA

**Fig. 2:**
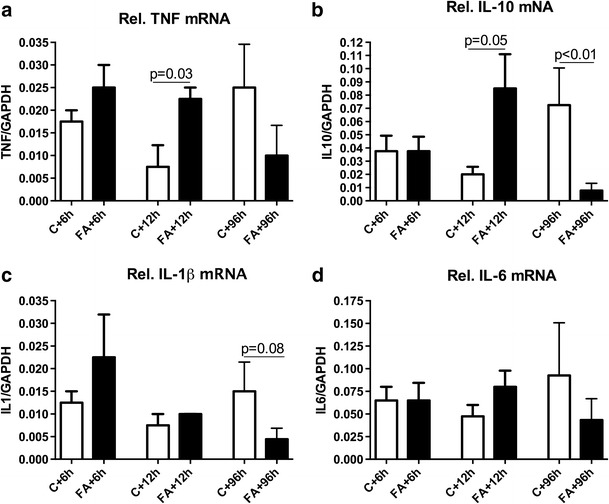
Increased TNF-α and IL-10 mRNA levels 12 h after fetal asphyxia, but downregulated cytokine levels at 96 h post-FA. Prenatal relative mRNA levels of TNF-α (**a**), IL-10 (**b**), IL-1β, (**c**), and IL-6 (**d**) in control and FA animals. mRNA levels are relative to GAPDH. Data shown as mean + SEM

To investigate the cerebellar inflammatory response after FA preconditioning, we examined by RT-PCR the mRNA levels of four different cytokines at 6, 12, and 96 h post-FA (Fig. 1). We found that both TNF-α (*p* = 0.03; Fig. 2a) and IL-10 (*p* = 0.05; Fig. 2b) were upregulated 12 h post-FA. mRNA levels for all cytokines tended to decrease 96 h after FA (Fig. 2a–d), while the decrease in IL-10 levels was significant compared to control levels (*p* < 0.01).

### Decreased Postnatal Cytokine mRNA Levels in Preconditioned Animals

**Fig. 3:**
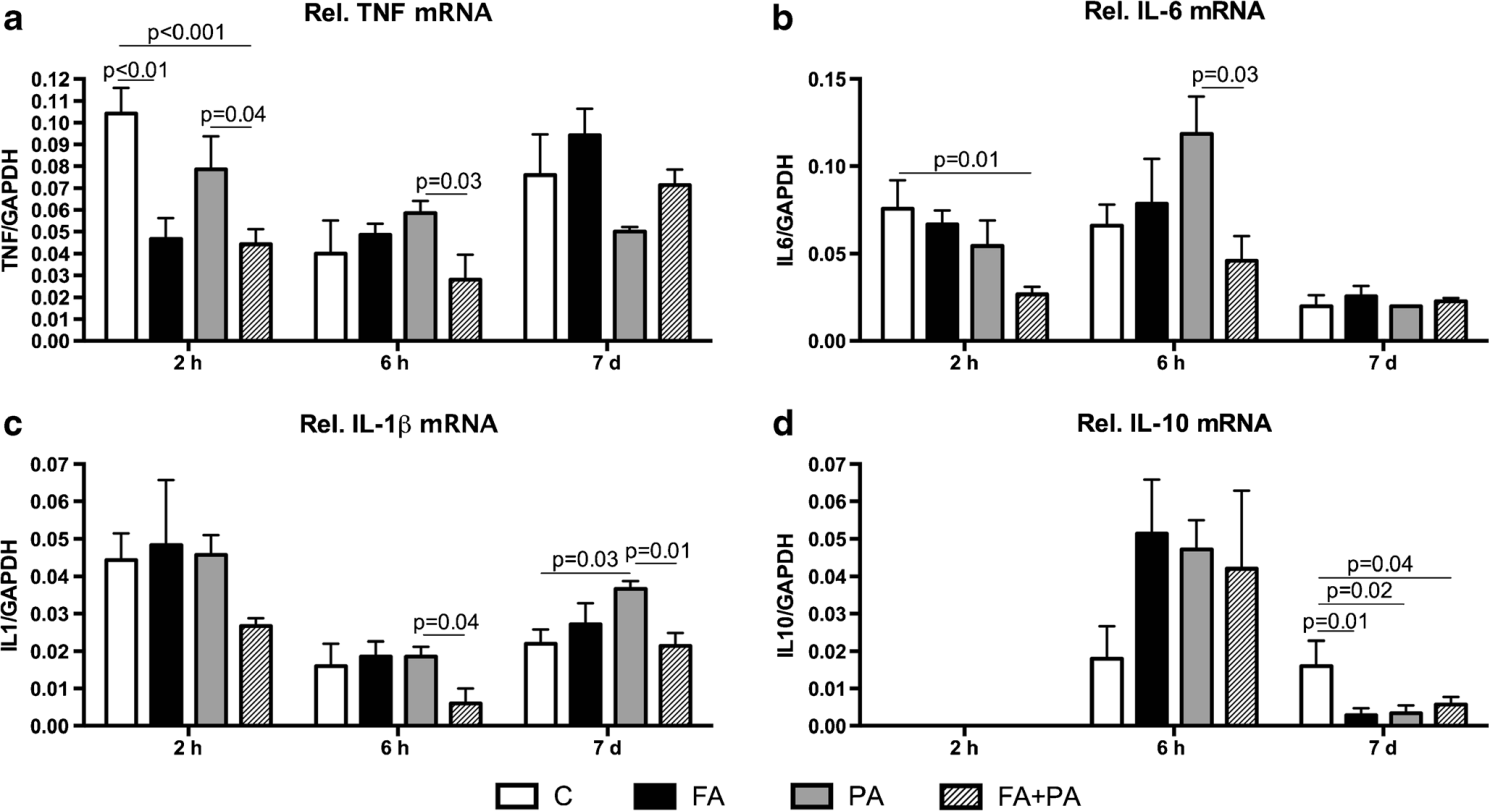
Downregulated cytokine mRNA levels in preconditioned animals. Postnatal relative mRNA levels of TNF-α (**a**), IL-6 (**b**), IL-1β (**c**), and IL-10 (**d**) in control, FA, PA and FA-PA animals. mRNA levels are relative to GAPDH. Data shown as mean + SEM

To investigate whether the changes in cytokine mRNA levels after sublethal FA would have any protective effect when combined with PA, we assessed cytokine levels at 2 h, 6 h, and 7 days after birth (Fig. 1). At 2 h after birth, TNF-α (*p* < 0.001) and IL-6 (*p* = 0.01) mRNA levels were lower in preconditioned animals (FA + PA group) compared to control animals (Fig. 3a, b). No mRNA levels could be observed for IL-10 in all groups (Fig. 3a). At 6 h after birth, preconditioned animals showed lower cerebellar levels of TNF-α (*p* = 0.03), IL-6 (*p* = 0.03), and IL-1β (*p* = 0.04) than PA animals (Fig. 3a–c). Even 7 days after birth, this effect was sustained for mRNA levels of IL-1β (Fig. 3c); with increased levels after PA (*p* = 0.03) but decreased IL-1β mRNA in FA-PA animals (*p* = 0.01). A different expression pattern was observed for mRNA levels of IL-10 (Fig. 3d). At 7 days after birth, IL-10 levels were decreased in all experimental groups compared with control animals.

### Cerebellar Cytokine Localization Based on Immunohistochemistry

**Fig. 4:**
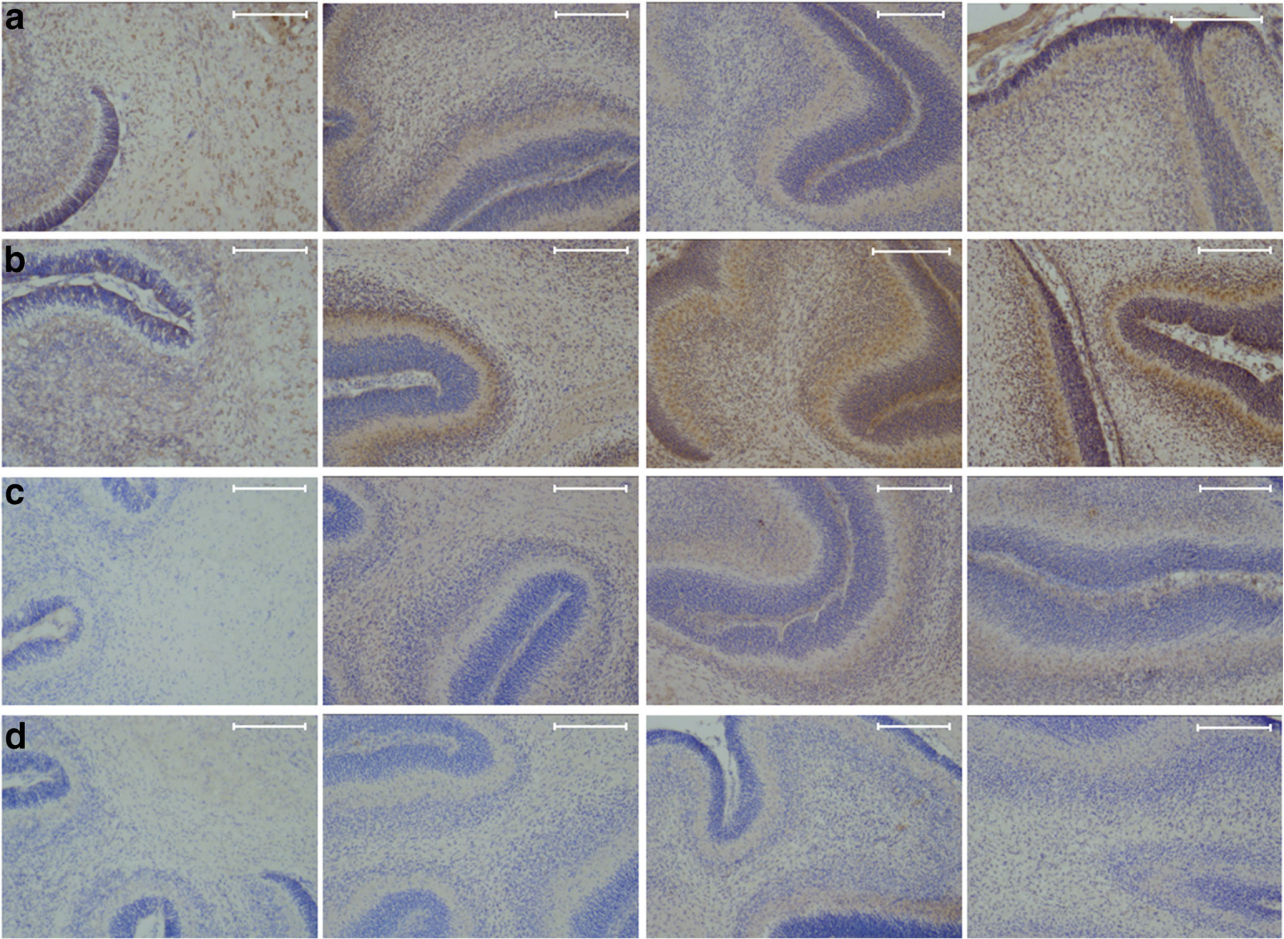
Cerebellar cytokine localization based on immunohistochemistry. Representative pictures (×10 magnification) of immunohistochemical staining of TNF-α (**a**), IL-1β (**b**), IL-10 (**c**), and IL-6 (**d**) of control (first column), FA (second column), PA (third column), and FA-PA (fourth column) animals 7 days after birth. Cytokine immunoreactivity is visualized with DAB (*brown*) and counterstained with cresyl violet. *Scale bars* 200 μM

To gain more detailed information concerning cytokine expression and localization, we performed immunohistochemical staining of TNF-α, IL-10, IL-1β, and IL-6 in postnatal rat brains. However, no clear differences between groups could be observed at 2 and 6 h after birth concerning the immunohistochemical cytokine staining (data not shown). Also at P7, when cerebellar structure is near mature [13], no clear differences in cytokine expression could be observed comparing control and asphyctic animals (Fig. 4a–d).

**Fig. 5:**
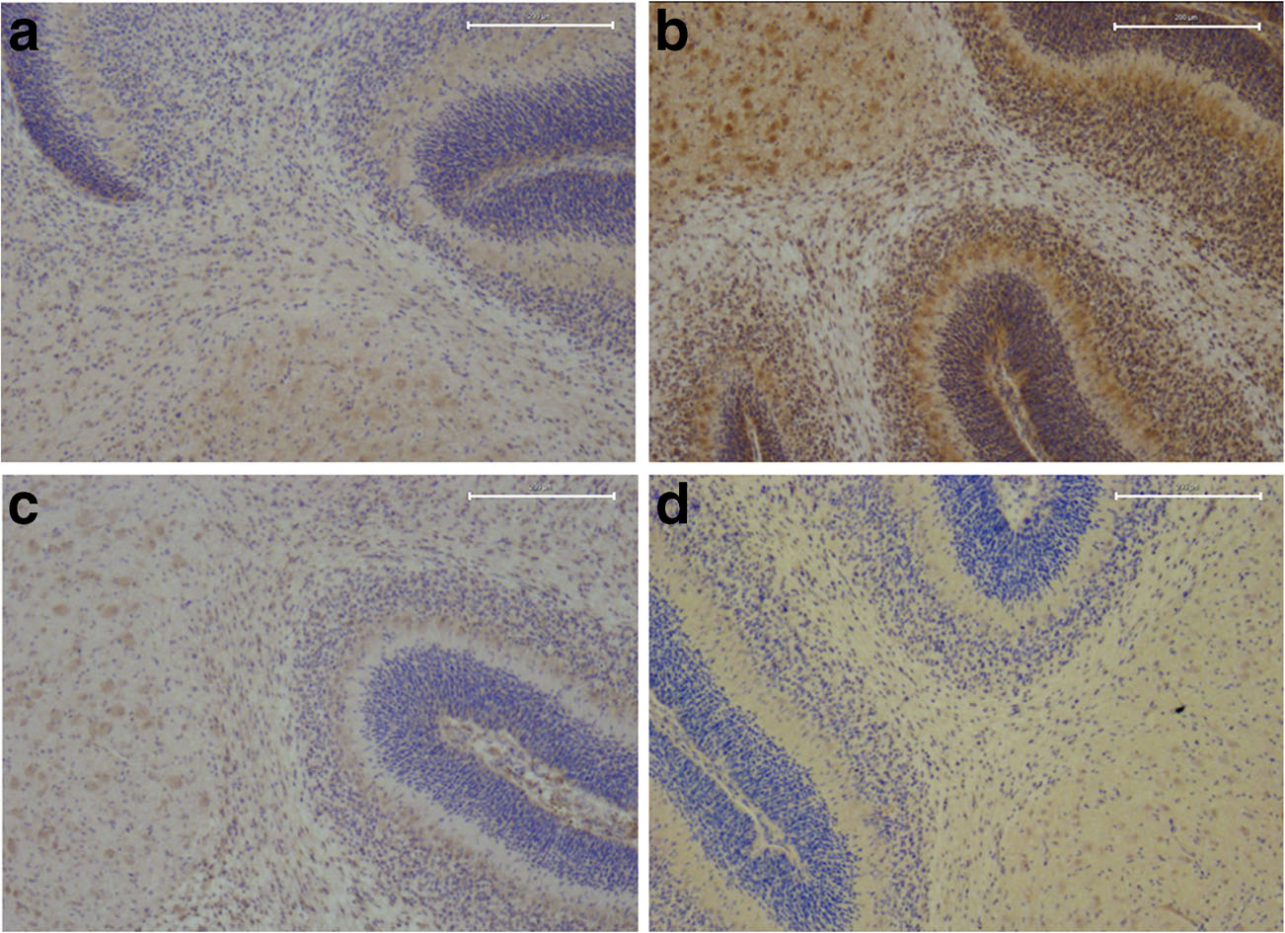
Cerebellar cytokine localization based on immunohistochemistry. Representative pictures (×10 magnification) of immunohistochemical staining of cerebellum at P7 of TNF-α (**a**), IL-1β (**b**), IL-10 (**c**), and IL-6 (**d**). Cytokine immunoreactivity is visualized with DAB (*brown*) and counterstained with cresyl violet. *Scale bars* 200 μM

**Fig. 6:**
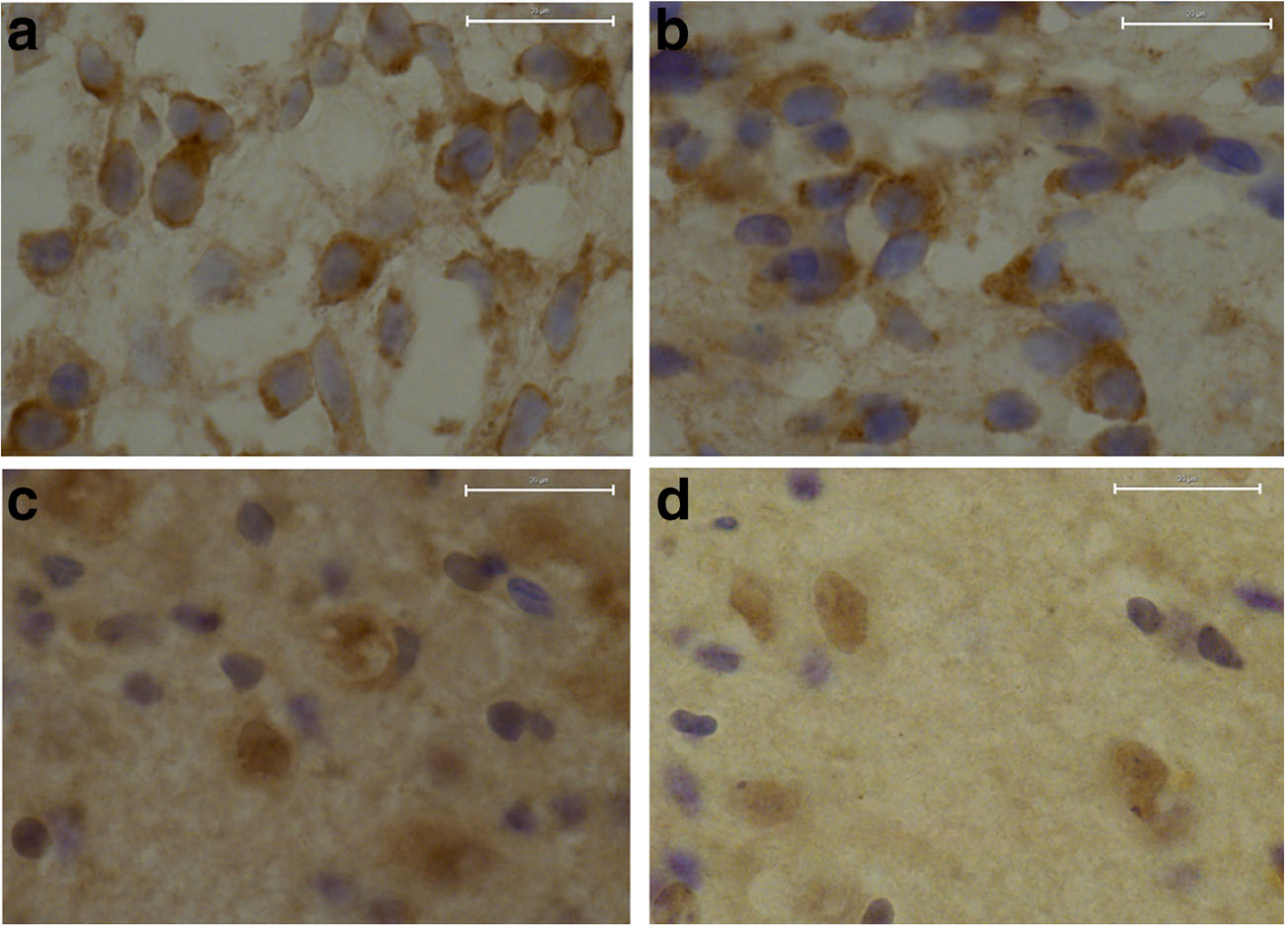
Diverse localization of measured cytokines in P7 brains. ×100 magnification of granular cells at P7 stained for TNF-α (**a**), IL-1β (**b**), IL-10 (**c**), and IL-6 (**d**). Cytokine immunoreactivity is visualized with DAB (*brown*) and counterstained with cresyl violet. *Scale bars* 20 μM

Overall, immunohistochemical staining of P7 rats clearly showed that TNF-α, IL-10, IL-1β, and IL-6 are expressed throughout the cerebellum with the highest cytokine immunoreactivity in the granular cell layer (Fig 5a–d). IL-β is also highly expressed in the cerebellar Purkinje cells (Fig. 5b). On the magnified pictures of the granular layer, we observed that TNF-α and IL-1β are mostly localized in the cytosol of the granular cells (Fig. 6a, b), while IL-10 expression was observed both in the nucleus and the extracellular matrix (Fig. 6c). Although IL-6 was only weakly expressed, staining revealed that this cytokine had comparable characteristics with IL-10; showing both nuclear and extracellular expression (Fig. 6d).

## Discussion

According to our knowledge, no studies have elucidated the inflammatory changes in the cerebellum after global fetal asphyctic preconditioning and global perinatal asphyxia. In this study, we demonstrate that fetal asphyxia per se downregulates the inflammatory cytokine responses in the cerebellum at time of birth when a perinatal asphyctic episode occurs. Further, cytokine levels are decreased up to postnatal day 7 when fetal asphyxia is followed by a perinatal insult, supporting the hypothesis that preconditioned animals are protected from asphyxia-induced cerebellar damage.

The current study is a follow-up on our previous work where we have shown that FA induces prenatal time-dependent cytokine responses in total brain [10]. The results indicated that these responses initiated with decreased cytokine levels after FA, while at 96 h post-FA the cytokine levels were increased. In contrast with the previous study, we here found increased TNF-α and IL-10 mRNA expression at first followed by a decrease in the cytokine levels at the time point immediately before birth. The increase in cytokine mRNA levels observed acutely after FA is not surprising. Many studies have highlighted that asphyxia induces inflammatory responses [6, 12, 14, 15]. It seems that in our study, the cerebellum can cope with the inflammation and is able to attenuate this response later on since the inflammatory cytokine responses are decreased at 96 h post-FA. The attenuation in inflammatory responses is most likely attributed to the effect of corticosteroids. It has been shown that the adrenal endogenous corticosteroids cope with stressful stimuli and subsequently downregulate inflammation [16, 17]. As at 96 h post-FA, the pups are primed to be born, we hypothesize that these post-asphyctic pups produce more corticosteroids, protecting themselves against further insults and corresponding damage. This might also explain why the preconditioned animals (FA + PA group) show a decrease in cytokine mRNA levels during postnatal life. Another explanation for the decreased inflammation after FA and thus the induction of neuroprotection when combined with PA could be attributed to the role of the mTOR pathway. During cerebral ischemia, the Akt/mTOR pathway is activated in response to hypoxia and mediates activation or stabilization of hypoxia-inducible factor-1 alpha (HIF-1) [18, 19]. Studies have shown that HIF-1 plays a neuroprotective role in rat brain with moderate ischemia–reperfusion via regulating apoptosis [18, 20, 21]. Hence, it is tempting to speculate that HIF-1 is activated in our model, leading to anti-apoptotic regulators and thus downregulating the inflammatory response. Further studies measuring corticosteroid and HIF-1 levels should be performed to better understand the underlying protective mechanisms.

In our studies, a global asphyctic insult is induced suggesting that forebrain and cerebellum are equally affected and that both will provide a similar response to the insult. Accordingly, we expected to observe high cytokine levels after the PA insult as we observed in total cerebrum at 2 and 6 h after the PA insult [10]. Surprisingly, we did not observe increased cytokine levels in the cerebellum due to PA alone at the acute time points after birth. Seven days after birth, however, increased levels of IL-1β were observed in PA animals. Dell’Anna and coworkers showed that cerebellar neuronal apoptosis in response to global asphyxia was maximal at postnatal day 8 [19]. Therefore, we assume that the cerebellum shows a delayed inflammatory response to PA compared to the rest of the brain due to the numerous neural connections between the forebrain and cerebellum. One major source of excitatory activation in the cerebellum is from the contralateral frontoparietal cortex via the corticopontocerebellar tracts. Functional disconnection of these transneural pathways likely underlies the phenomenon of crossed cerebellar diaschisis, in which remote cerebral injury results in cell loss and atrophy of the contralateral cerebellar hemisphere [22]. Many forebrain neurons also connect via the ipsilateral cerebellum [23]. Thus, damage to the forebrain may lead to secondary damage in the ipsilateral cerebellum and then to tertiary damage in the contralateral hemisphere [24]. Studies using animal models for perinatal hypoxia–ischemia have indeed shown that delayed cell death occurs in brain regions that are not directly affected by the ischemia, such as cerebellum. Accordingly, it seems that in the immature brain neuronal connectivity does participate in the observed neurodegeneration after hypoxia–ischemia [13, 25].

At time of birth the cerebellum is not fully developed. Due to the nearly fivefold increase in growth in the cerebellum in the last trimester of pregnancy [5, 22], activation of the fetal immune system can cause irreversible damage to this structure [26]. It is well documented that the cerebellum is involved in motor functions such as coordination, posture, and equilibrium [4]. Moreover, in preterm infants or in the neonatal period, cerebellar injury has been implicated in cognitive-behavioral dysfunction [13, 27, 28]. Previously, we have shown that PA induces motor impairments and that FA as preconditioning stimulus can protect against these PA-induced locomotor and cognitive problems [8]. Here, the present findings reveal that preconditioned animals show lower cerebellar cytokine levels after perinatal asphyxia, suggesting that this can be a part of the mechanisms of permanent neuroprotection of sublethal fetal asphyxia.

In conclusion, we show that cerebellar inflammation contributes to the pathogenesis of asphyctic insults and disclose part of the potential molecular targets for perinatal anti-inflammatory therapy. Which specific anti-inflammatory therapy is the most appropriate cannot be answered at this time. However, it is clear that the present data encourage research to test the applicability of anti-inflammatory therapy to the perinatal clinical setting.
